# Novel automated method to assess group dynamics reveals deficits in behavioral contagion in rats with social deficits

**DOI:** 10.3389/fnbeh.2024.1519486

**Published:** 2024-12-18

**Authors:** Kirill Smirnov, Ilya Starkov, Olga Sysoeva, Inna Midzyanovskaya

**Affiliations:** ^1^Institute of Higher Nervous Activity and Neurophysiology, Russian Academy of Sciences, Moscow, Russia; ^2^Faculty of Computer Science, National Research University Higher School of Economics, Moscow, Russia; ^3^Center for Cognitive Sciences, Sirius University of Science and Technology, Sochi, Russia

**Keywords:** automated behavioral testing, IntelliCage, behavioral contagion, social deficit, rat, autism spectrum disorder, group behavior

## Abstract

Behavioral copying is a key process in group actions, but it is challenging for individuals with autism spectrum disorder (ASD). We investigated behavioral contagion, or instinctual replication of behaviors, in Krushinky-Molodkina (KM) rats (*n* = 16), a new potential rodent model for ASD, compared to control Wistar rats (*n* = 15). A randomly chosen healthy Wistar male (“demonstrator rat”) was introduced to the homecage of experimental rats (“observers”) 10–14 days before the experiments to become a member of the group. For the implementation of the behavioral contagion experiment, we used the IntelliCage system, where rats can live in a group of 5–6 rats and their water visits can be automatically scored. During the experiment, the demonstrator was taken out of IntelliCage for a pre-test water deprivation and then placed back for the behavioral contagion test. As a result, a drinking behavior of the water-deprived demonstrator rat prompted water-seeking and drinking behaviors in the whole group. Unlike the Wistar controls, KM observers showed fewer visits to the drinking bottles, particularly lacking inspection visits (i.e., visits without drinking). The control group, in contrast, exhibited a dynamic, cascade-like visiting of the water corners. The proportion of activated observers in KM rats was significantly lower, as compared to Wistar ones, and they did not mimic other observer rats. KM rats, therefore, displayed an attenuated pattern of behavioral contagion, highlighting social deficits in this strain. This study suggests that measuring group dynamics of behavioral contagion in an automated, non-invasive setup offers valuable insights into social behavior in rodents.

## 1 Introduction

Behavioral copying is the simplest mode of social learning where a mood, attitude, or behavior spreads quickly in social groups (Polansky et al., 1950). This phenomenon enhances collective coordination and fosters social cohesion, also reducing mutual aggression (Laméris et al., 2020). However, this mode of behavior is deficient in individuals with autism spectrum disorder (ASD) (Stewart et al., 2013). The decreased ability to replicate the behavior of others facilitates failures in social or academic training (Pittet et al., 2022) necessitating tutorial support for ASD pupils.

The development of animal models for ASD is crucial for advancing treatment strategies. They allow experimental approaches to study social deficits and, in translational perspectives, lead to effective support for patients (Chadman et al., 2012; Ruhela et al., 2015; Sierra-Arregui et al., 2020; Silverman et al., 2022) .

One promising approach is screening existing animal models of epilepsy for ASD phenotypes, as these two conditions are frequently comorbid in clinical populations and may share common pathobiological mechanisms. Specifically, the prevalence of epilepsy in individuals with ASD is 13% (range: 2–60%), while ASD is diagnosed in 9% (range: 1–42%) of individuals with epilepsy (Lukmanji et al., 2019). Recently, we hypothesized that the Krushinky-Molodkina (KM) rat strain could model a specific subtype of ASD: ASD with comorbid latent epilepsy but without Attention Deficit Hyperactivity Disorder (Rebik et al., 2022). The KM rat strain was started in the 1950s at the Biological Faculty of Moscow State University by Leonid Krushinsky, who initially observed a Wistar rat which developed seizures in response to environmental sounds, such as the ringing of keys. Descendants of this rat were selectively bred over 90 generations for maximal predisposition to audiogenic seizures, ultimately resulting in a fully inbred strain with nearly 100% seizure susceptibility (Poletaeva et al., 2017). Upon sound stimulation, these rats exhibit tonic seizures with a latency of less than 10 s. The susceptibility to audiogenic seizures in KM rats is polygenic, with most alleles conferring susceptibility being recessive (Romanova et al., 1993). Although phenotypic seizures are not typically observed until 6–8 weeks of age, an excitatory/inhibitory imbalance may already be present in early ontogeny, as neural progenitor cells in KM rats are predominantly glutamatergic (Naumova et al., 2020) Neuroinflammatory signs have been documented in KM rats (Chuvakova et al., 2021), and brain cytokines are activated by audiogenic seizures, potentially mediating epileptogenesis (Surina et al., 2023). In addition, KM rats exhibit imbalanced binding to D1-like and D2-like dopamine receptors in the insular cortex (Birioukova et al., 2024), a region implicated in ASD pathology (Blum et al., 2024; Mittleman and Blaha, 2015; Sato et al., 2023). KM rats also show learning difficulties, e.g., slow avoidance learning (Surina et al., 2024), and exhibit anxiety traits (Surina et al., 2011).

We hypothesized that engagement in collective actions would be more difficult for KM rats compared to healthy Wistar rats. In several experimental paradigms—including the 3-chambered and 2-chambered social preference/social novelty tests, as well as the socially enriched open field (Rebik et al., 2023) test—KM rats exhibited fewer approaches to the encaged social stimuli (which were unfamiliar outbred Wistar males of similar age and weight). Additionally, the proportion of prolonged (> 6 s) visits compared to short (< 6 s) visits was lower in KM rats than in the Wistar controls. However, when the social stimuli were replaced with inanimate novel objects placed in the same experimental setup, KM rats interacted more frequently with the objects. These findings suggest that social interaction may be aversive for KM rats, and they appear to lack motivation for social contact. Surprisingly, KM rats did not lose in the social dominance tube test, but they consistently outperformed the Wistar rats. This outcome was likely due to a limited behavioral repertoire in the KM rats, who mostly displayed passive behaviors, such as sitting in place or moving slowly forward, without attempts to push, groom their opponent, or retreat from the tube (Rebik et al., 2023).

**FIGURE 1:**
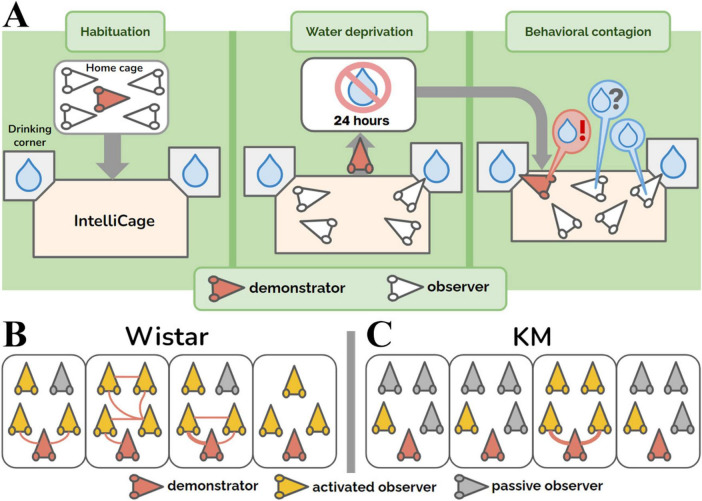
Group aspects of behavioral contagion. **(A)** Experimental design. **(B,C)** Group graphs showing the dynamics of contagion in the cohorts of control Wistar (panel **B**) and KM (panel **C**) rats. The group graphs represent 30 min of observation. Red rat heads indicate the demonstrator rats (i.e., water-deprived animals) in each group. Yellow rat heads represent the activated observer rats, i.e., non-deprived animals that made at least one visit to the water corner. Gray rat heads correspond to passive observers, which did not visit the water corners. Red edges between the nodes represent quickly (< 4 s) copied visits to the water corner, as demonstrated by any groupmate. The thickness of the lines is proportional to the number of rapidly copied visits (ranging from 0 to 3 in this study).

Subjects with an autistic-like phenotype may be particularly sensitive to environmental stressors, including novel surroundings or inescapable handling. To reduce anthropogenic stress and novelty-induced environmental dishabituation, we adapted the behavioral contagion paradigm for dyadic interaction in rodents (Ivanov et al., 2014). In those studies, a demonstrator rat, that was isolated and water-deprived for 24 h, performed extensive drinking upon reintroduction into the cage with a conspecific and this behavior was replicated by the non-deprived observer rat (Ivanov et al., 2014; Ivanov and Krupina, 2017). This phenomenon was absent in the case of unmotivated (i.e., not water-deprived) demonstrators (Ivanov et al., 2014). In this study, we modified the paradigm of behavioral contagion for automated testing in a group of rats. For this purpose we used the automated behavioral monitoring system IntelliCage, which allows to score automatically the drinking behaviors of rats in a social environment without the interference of handling by the experimenters (d’Isa and Gerlai, 2022; Kiryk et al., 2020; Lipp et al., 2024; Voikar and Gaburro, 2020). Rats were tested in social groups of 4–5 individuals. The drinking bottles were located in corner compartments, where access to water was limited by automated opening and closing of metal doors. This setup allowed for the identification of each individual animal inside the drinking corners. The demonstrator rat, which had been a group member for at least 10–14 days in the standard home-cages before all the cage-mates entered the IntelliCage, was isolated and water-deprived for 24 hours (Figure 1A). As expected, soon after reintroduction, the thirsty demonstrator rat started drinking, while the intact and non-deprived observer rats behaved freely and could copy or not copy the water corner visits. Our study reports the behavioral parameters of observer rats tested in this situation and shows an attenuated contagion response in KM rats, which suggests an impairment in their social cognition (Rebik et al., 2023; Rebik et al., 2022).

## 2 Materials and methods

### 2.1 Animals

The experiments enrolled intact adult male rats, aged 6–8 months and weighing 350–450 g. The cohorts were 15 outbred Wistar rats and 16 inbred KM rats. The animals arrived at the animal facility of the Institute of Higher Nervous Activity and Neurophysiology at the age of 6–8 weeks. The rats were housed 4–5 per cage (53 × 34 × 17 cm, L × W × H), with *ad libitum* access to standard food (chow pellets by Gatchinsky Feed Mill, Malye Kolpany, Russia) and tap water, under a 12-hour light/dark cycle (lights on at 8:00 A.M.) A month prior to the main experiment, all the rats were implanted with individual identification veterinary chips veterinary chips (passive radiofrequency identification transponders), below the skin and above the neck muscles, under mild sedation (dexmedetomidine 0.2 mg/kg, i.m.). A new, randomly chosen and previously unfamiliar Wistar male rat, of the same age and weight, was added to each group of 3–4 rats, 10–14 days prior to the main experiment. It served as the demonstrator later. The given time period of common housing is sufficient for the albino rat to display pro-social behavior toward rats of the other strain (Ben-Ami Bartal et al., 2014). After the given period, the cage-mates were considered as a group in our further experiments.

### 2.2 IntelliCage set-up

We used the IntelliCage set-up with two available drinking corners (TSE Systems GmbH, Germany).^1^ The size of the central arena was 50 × 100 × 35 cm, with wood shavings as bedding material, and two standard gray plastic shelters (TSE Systems, Bad Homburg, Germany), allowing the animals to hide and climb. The pre-implanted veterinary chips allowed to collect individual information on the entries, occupation time and number of lickings made inside the water corners.

**FIGURE 2:**
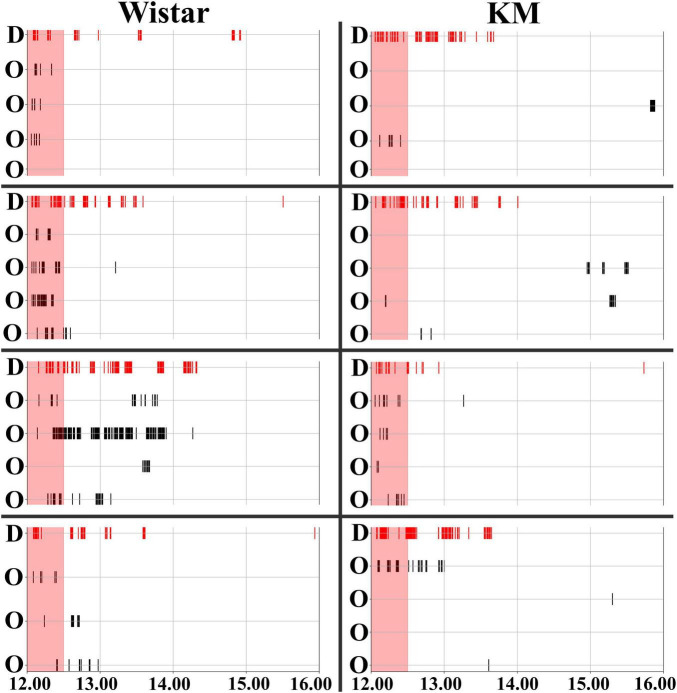
Timeline of behavioral contagion in Wistar and KM Rats. Water corner visits were recorded for Wistar (left panels) and KM (right panels) rats. The timeline shows activities over a 4-hour period, starting from the re-introduction of the demonstrators. The contagion period, marked by red shading, is the period analyzed in the study (the first 30 min). Visits to the water corner by the demonstrators (denoted by “D” on the left side of each individual group chart) are indicated by red vertical stripes at the top of the chart. Visits by the observer rats (denoted by “O” on the left side of each individual group chart) are shown with black vertical stripes. The thickness of the lines is proportional to the duration of time each individual rat spent in the water corner.

The experiment consisted of three blocks (Figure 1A). All the procedures (the start of all blocks, water deprivation protocols, and re-introduction of the individual demonstrator rats) were set up at 12.01 PM. The first three days were the habituation period: the doors to the drinking bottles opened every time a rat was detected inside. The drinking time was unlimited. Subsequently, the rats were trained to open the doors by poking their noses into a special area inside the corners. The door opening time was 15 s, after which the rat had to re-entry in order to access water again. As all the rats habituated to the procedure, the water-deprivation procedure started for the demonstrator. The demonstrator rat was withdrawn from the group, put in its home cage without water available, and the cage was placed in another room for 24 h. All the observer rats left in the IntelliCage had the same conditions of water access, as earlier. For the behavioral contagion test, the water-deprived demonstrator rat was returned to the IntelliCage. The activity registration lasted for at least 4 hours. The deprived rat demonstrated the most pronounced drinking behavior within the first hour (Figure 2) after re-introduction. The time interval of 30 min was chosen for the main behavioral analysis based on an overview of 4 hours activity (Figure 2).

### 2.3 Experimental registration

Each water corner was equipped with sensors capable of detecting the veterinary chips of the rats, and the drinking bottles were endowed with lick contact time sensors. The recorded parameters included the rat ID, the times of entry and exit, and the number of licks made during each visit to the water corner. Data were collected throughout the entire period the rats were housed in the IntelliCage apparatus. To assess natural circadian variation in the rats’ activity, we analyzed the third day of the habituation session (two periods, from 9:00 to 12:00 a.m. and from 12:00 to 3:00 p.m.). On the day of the behavioral contagion test, the time period from 8:50 to 11:50 a.m. was used to establish a baseline of activity immediately prior to the presentation of the social stimulus. Finally, contagion effects were assessed during the 30 min following the re-introduction of the demonstrator rats. All data were time-normalized: the three-hour baseline data sets were divided by 6 to match the duration of the 30-min contagion period (see Supplementary Table 1).

### 2.4 Graph construction

The collective behavior was presented as a graph, built up for the 30 min of behavioral contagion, for each individual group (Figures 1B, C). The following definitions were employed:

• For each graph, the nodes represent individual rats, linked by edges (the connections between the nodes), and the thickness of the red edges indicates the association index (i.e., the number of copied visits, as shown in Figures 1B, C).
• A water corner was considered as demonstrated, immediately after a visit of any rat.
• The visit was regarded as a copied one (red lines as the graph edges, Figure 1B), if another rat entered the demonstrated drinking corner with a latency < 4 s. The threshold was modified from a previous work (Ivanov et al., 2014), where it was 2 s. Here we enlarged the latency of copied visits due to a complex geometry of the IntelliCage’s drinking corners, with their tunnel-like entrance chambers, preceded by steps.
• An observer rat was considered ‘activated’ and depicted as a yellow rat head (Figures 1B, C) if it made at least one visit to any water corner during the 30-min period of contagion, regardless of whether the visit was subsequently copied again by a following rat. If no such visit occurred, the rat was classified as a ‘passive observer’ and depicted as gray rat head (Figures 1B, C).

The duration of captured interactions ranged from 4 s to 30 min, allowing us to assess the full dynamics of contagion (see Supplementary Table 2).

### 2.5 Statistics

In this study, we first conducted a nested ANOVA, with the “cage” factor nested within the “strain” factor, to assess between-strain differences. Next, we performed repeated measures ANOVAs for each strain, using time conditions (baseline and contagion) as the within-subjects factor and “cage” as the between-subjects factor, to examine contagion effects and their dependence on collective traits within each cage group. The following parameters were analyzed:

1. Total number of visits
2. Number of drinking visits
3. Number of inspection visits (i.e., without drinking)
4. Total licking time

As an additional analysis, potential circadian shifts between key time points were assessed using baseline habituation data, by repeated measures ANOVA. Specifically, on the third day of the habituation session, activity during the 9:00–12:00 morning hours was compared to activity during the 12:00–15:00 midday hours to examine general activity patterns. The “strain” was taken as between factor, and “time” was considered as a within factor.

Correlations between different metrics were assessed using Spearman’s rank correlation. Specifically, baseline licking duration, number of drinking entries, and inspection entries were correlated with the same parameters measured during the 30-min contagion period. This approach allowed us to explore whether pre-contagion (baseline) activity influenced individual responses during contagion.

The assessment of collective activity was carried out on the basis of individual group graphs (Figures 1B, C). The proportion of active (yellow rat heads, see 2.4) observers was compared between the Wistar and KM cohorts using Chi-square test (Tables 2*2). The same was done for the proportion of observer-observer copying: the actual number of red edges between yellow “rat head” signs of activated observers, referring to the maximum possible number of all edges (which is (N^2^-N)/2, where N is the group size). The group parameters (the number of actual edges and the number of all possible edges) are summarized for each strain and compared using Chi-square test.

Statistica 12.0 software was used for the analysis, along with custom-written Python scripts to construct and visualize graphs.^2^

## 3 Results

### 3.1 Baseline activity of Wistar and KM rats

Baseline behavior was assessed over a 3 h period from 8:50 to 11:50. This time window began 50 min after the lights were turned on, allowing the rats to acclimate to the change, and ended 10 min before the main contagion block. A 3 h duration was chosen due to the low level of spontaneous activity during the light phase; a shorter period would have introduced greater random fluctuations in the data. No significant differences were found between the strains in terms of the baseline number of active animals: 10 out of 15 Wistar rats and 7 out of 16 KM rats visited the water corners (Chi-square = 1.643, *p* = 0.199). Additionally, there were no significant differences in the total number of visits {*F*(1, 30) = 8.97, *p* = 0.19}, the number of drinking visits {*F*(1, 30) = 5.26, *p* = 0.29}, or the duration of drinking {*F*(1, 30) = 2.99, *p* = 0.78}.

To account for potential circadian shifts associated with the experimental time points, an additional analysis was conducted. Morning spontaneous visiting activity (9:00–12:00) was compared to midday activity (12:00–15:00) in two rat cohorts (KM and Wistar) on the third day of the habituation period. No significant strain differences were observed (*p* = 0.86), while the daytime factor showed only a tendency toward significance (*p* = 0.06): 0.6 ± 0.9 visits per 30 min during the morning (9:00–12:00) and 0.3 ± 0.6 visits per 30 min during the midday period (12:00–15:00). Therefore, circadian shifts do not appear to favor the contagion effect.

### 3.2 The behavioral contagion phenomenon

Behavioral contagion was characterized by a sequence of simple actions, such as water corner visits and water consumption, performed by the rats. The observer rats, which were non-deprived, either responded to or did not respond to the demonstrator’s drinking behavior. Observer rats’ responses (water corner visits) are marked by black vertical stripes in each chart of Figure 2, while the activity of the demonstrator rat is indicated by red vertical stripes. All demonstrator rats initiated their serial drinking visits upon reintroduction to the IntelliCage (Figure 2), continuing to shuttle between the available water bottles for an extended period (Beck, 1962), as shown by the experimental timelines (red lines in each chart of Figure 2). A 30-min time interval was used for the analysis of behavioral contagion, which is highlighted by the red shading in Figure 2. This interval was selected based on visual inspection of the of the wider 4 h recording time window, where a clear pattern of contagion activity was observed within the first 30 min.

### 3.3 Strain and group differences in behavioral contagion

The control rats exhibited dynamic engagement in behavioral contagion (Figures 1B, 2 left panel), which was poorly observed in KM rats (Figures 1C, 2 right panels). Specifically, the proportion of activated observers (i.e., the observer rats which made at least one visit to any water corner during the 30 min of contagion period, marked as the yellow rat heads on Figures 1B, C) was significantly lower in the KM cohort (7/16 in KM rats vs. 13/15 in Wistar rats; Chi-square = 6.229, *p* = 0.012). Additionally, the proportion of followers (i.e., the group mates which quickly replicated at least one visit, red links in Figures 1B, C) was also significantly lower in KM rats. Specifically, 2 out of 16 KM observers copied visits to the demonstrated water corners, compared to 8 out of 15 Wistar observer rats (Chi-square = 5.907, *p* = 0.016). In other terms, KM rats actualized 2 out of 38 possible edges within their 4 groups, and Wistar rats actualized 10 out of 36 possible edges within their 4 groups (Chi-square = 7.393, *p* = 0.007 for the strain difference).

**FIGURE 3:**
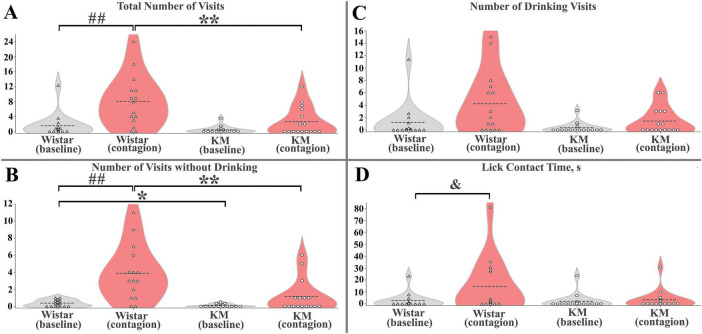
Quantitative parameters of behavioral contagion in Wistar and KM rats. **(A)** Total number of visits **(B)** Number of inspection (without drinking) visits. **(C)** Number of drinking visits. **(D)** Licking time, in seconds. For panels **(A–D)**, the significance level was set at *p* < 0.05. Between-strain differences are marked with “*”; contagion effects (test vs. baseline) are indicated by “##”; and cage × contagion interactions are shown with “&”. A single symbol represents *p* < 0.05, two symbols represent *p* < 0.01. See the Results section for more details.

Wistar and KM observers differed in their responses to the reintroduced demonstrators, with the variation partly dependent on a collective trait specific to each group of cage-mates. Under baseline conditions, the “cage” factor, nested within the “strain” factor, was not significant in any respect; a moderate strain difference was observed only for inspection visits {*F*(1, 30) = 5.4, *p* = 0.03; Figure 3B}, which corresponds well with a slightly decreased investigatory activity found in rats with the valproate syndrome, a validated animal model for ASD (Pelsűczi et al., 2020). During the behavioral contagion phase, the strain difference remained highly significant for this behavior {*F*(1, 30) = 8.5, *p* = 0.007; Figure 3B}, again independent of the “cage(strain)” factor. The total number of observers’ visits during the contagion phase also showed a significant strain difference, independent of the “cage(strain)” factor {*F*(1, 30) = 8.6, *p* = 0.007; Figure 3A}. Under contagion conditions, drinking visits and total licking time also demonstrated a strain difference {*F*(1, 30) = 3.0, *p* = 0.03 and *F*(1, 30) = 4.6, *p* = 0.003, correspondingly}, along with a significant “cage(strain)” effect {*F*(6, 30) = 3.0, *p* = 0.03 and *F*(6, 30) = 4.6, *p* = 0.003, respectively]. These findings suggest that collective traits may play an important role in contagion-induced consumption. To explore this further, contagion was analyzed separately within each strain, accounting for repeated measures within samples and potential “cage” effects.}

Within each strain, behavioral contagion led to some facilitation of visiting activity (Figures 2, 3). This effect was not significant in the KM group but was prominent in the Wistar sample. Specifically, none of the measured parameters showed significant differences between baseline and contagion conditions in KM rats, and also the “cage” factor did not have a significant impact.

In contrast, in the Wistar sample, significant effects were observed both independent of the “cage” factor and with notable variation between the groups of cage-mates. Specifically, universal (not cage-dependent) responses included the total visits and inspection (without drinking) visits, with significant effects: total visits *F*(1, 14) = 12.6, *p* = 0.005, and inspection visits *F*(1, 14) = 18.4, *p* = 0.001 (Figures 3A, B). The variable response included total licking time, which showed a contagion effect {*F*(1, 14) = 5.2, *p* = 0.04} and a significant “cage” effect {*F*(3, 14) = 5.2, *p* = 0.02}, with a significant interaction between the two {*F*(3, 14) = 4.8, *p* = 0.02, Figure 3D}. A similar trend was observed for the number of drinking visits, where the contagion effect tended to be significant (*p* = 0.06), and the “cage” effect was significant {*F*(3, 14) = 3.6, *p* = 0.048}.

Behavioral contagion was not correlated with the baseline water consumption in observers of both rat strains (according to the Spearman rank order correlation, all *p*-values > 0.10), indicating the independence of the induced behaviors from putative drinking motivation. Additionally, no correlations were found between other baseline measures and the corresponding behavioral parameters (all *p*-values > 0.10).

To summarize, KM rats did not show a significant increase in water-seeking behaviors during the contagion period, as indicated by the low number of mimicked behavioral acts (Figures 3A–D) and fewer activated observer rats (Figures 1B, C). In contrast, Wistar observers exhibited an increased level of water-seeking behaviors following the reintroduction of the demonstrators, which was universally expressed as increased inspection visits in the water corners (Figure 3B) and, in some cohorts, elevated drinking (Figure 3D). The Wistar rats repeatedly entered the water corners, both following the demonstrators and other group members (Figures 1 B, C). These copied visits occurred immediately and with some latency during the contagion period. The variability in contagion-induced water consumption in healthy rats may be influenced by the collective traits of each cagemate cohort and could also result from random factors, such as a higher proportion of active animals within the group.

## 4 Discussion

### 4.1 Components of behavioral contagion

Engagement in collective behaviors is a basic survival mechanism in gregarious animals, essential for activities like social foraging and collective escape. In foraging, it is more efficient to join group success rather than making random trials. In this study, we observed that the simplest mode of group action— mimicking of a behavioral pattern —is well expressed in laboratory rodents, outbred neurotypical Wistar rats.

Behavioral contagion is a form of allelomimetic behavior that plays a role in the social cohesion (Ginelli et al., 2015). According to the social psychologist Ladd Wheeler, behavioral contagion should be distinguished from conformity, imitation, social pressures, and social facilitation (Wheeler, 1966). While “social conformity” and “social pressure” are challenging to assess in animal behavior, “imitation” (behavioral mimicking) and “social facilitation” can be objectively assessed in animal experimentation. Observer rats responded to demonstrators’ behaviors both by drinking and by inspecting water corners.

Further experiments are required to discriminate between imitation, goal emulation, and social facilitation as reasons for contagious behavior in laboratory rats. Notably, there was no correlation between the amount of water consumed during the baseline period, and the number of water corner visits in the behavioral contagion test. Therefore, intrinsic motivation to drink was not necessary for observers to copy the demonstrated pattern. This allows us to distinguish the observed phenomenon from social facilitation, i.e., the activation of motivated behavior by observing its execution in another subject (Redd and de Castro, 1992).

In the dyadic paradigm of behavioral contagion, thirsty demonstrators prompted observer rats to attend the water bottles more frequently, and the effect lasted 8 min (Ivanov et al., 2014). The ethograms of observer rats show a lack of aggressive behavioral elements toward the re-introduced water-deprived demonstrator rats, compared to the agonistic interactions observed with non-deprived demonstrators (Ivanov and Krupina, 2017). Here, we set up a group test that required extending the observation period because of a larger number of animals. Motivated behavior consists of sequences of actions organized within nested behavioral states (Sanabria et al., 2019). The modified behavioral contagion test allowed us to distinguish between visits made for water consumption and those made only for inspecting the bottles, making possible to dissect the distinct motivations underlying water-seeking behavior.

Social foraging implies that each animal monitors the foraging success of the group members to share any found resource. We observed that control rats were more likely to inspect visited water bottles rather than drink from them (Figures 3B, C), as if the action of the demonstrator caused in the observers primarily a shift of attention toward the water corners more than an increase of their motivation to drink. Notably, this behavioral contagion effect is possible only if the observer pays attention to the actions of the demonstrator. Active attention to the behavior of a group member is essential for social learning in gregarious animals. Attention to conspecifics’ behavior is, for instance, a prerequisite for contagious yawning, another model of neutral behavior that spreads in animal groups (Gallup, 2021).

### 4.2 Attenuated behavioral contagion in KM rats

As hypothesized, KM rats showed poor reactions to demonstrators’ behavior, with low individual responses and group engagement. This extends our previous findings on deficient social motivation in KM rats (Rebik et al., 2023; Rebik et al., 2022). The present setup allowed observer rats to behave freely, minimizing stress and providing an objective way to test social cognition deficits.

Attention plays a crucial role in the contagion-induced behavioral responses (Gallup, 2021, 2022). Rats with an ASD-like phenotype may pay less attention to social stimuli, and consequently to the demonstrated actions, resulting in minimal motor replication. Special training approaches improve motor imitation in children with ASD, aiding social coping (Paparella and Freeman, 2021). Motor imitation in early childhood enhances language abilities, while reduced observational learning in adults with ASD-like traits is linked to reduced goal emulation (Wu et al., 2024). Imitation attempts in adults with ASD might be a part of social camouflage (Alaghband-rad et al., 2023).

In rodents, social camouflaging is unlikely, but insufficient imitation abilities are detectable. In adult KM rats, deficiencies are seen at the level of behavioral imitation. Special experiments are needed to infer the ability of goal emulation in KM rats. Experimental manipulations that facilitate social learning in rodent models of ASD may serve as translational points for research in ASD neurobiology and treatment.

### 4.3 Group dynamics

Group aspects of the behavioral contagion test provide new insights into sociability in rats, which are highly gregarious animals (Latané, 1969). Rats sharing the same housing enclosures form non-random huddling associations (Proops et al., 2021). Despite over 70 years of research have been performed after the rodent sociability models of the mid-20th century (Davis, 1953), methodological limitations persist, as most experiments involve direct handling, removal of animals from their familiar environment, and short-term testing. Recent experimental systems that allow long-term, automated behavior tracking have emphasized the complexity and significance of group interactions in laboratory rats (Nagy et al., 2023).

Studies in schooling fish reveal that small groups of strongly connected individuals are both the most socially influential and the most susceptible to social influence (Rosenthal et al., 2015). In bird flocks, the phenomenon of murmuration demonstrates how the behavioral state of one bird influences, and is influenced by, all others (Cavagna et al., 2010). In avian studies, murmuration refers to the synchronized patterns of bird flying, with each bird adjusting its position based on its neighbors. This creates dynamic shapes in the sky and is thought to aid in predator protection and serve as a form of communication or social bonding. While individual actions within a group are inherently stochastic, understanding intrinsic group dynamics is crucial to comprehend behavioral contagion (Cavagna et al., 2010; Rosenthal et al., 2015).

The rat groups in this study were smaller than fish or birds flocks, but dynamic behavioral effects were still observed. In healthy Wistar rats, activated members served as demonstrators for others (Figure 1B), facilitating social action. Non-deprived observer rats replicated water corner visits, even with no need for additional drinking.

In contrast, the KM sample exhibited lower baseline investigatory activity (Figure 3B), similar to what was previously observed in a valproate-treated sample (Pelsűczi et al., 2020), as well as reduced socially mediated engagement (Figures 1C, 3A–D). KM group members did not mimic other cage mates. It is unclear whether the demonstrators failed to gain a high social rank in the KM cohorts during the preliminary common housing period. While one might hypothesize that low-ranked members are not followed, this should not be a strict rule for foraging-related behaviors like water consumption. Success sharing in foraging should be effective regardless of group rank (otherwise, it would decrease the chance to satiety). Additionally, KM observers did not copy each other within their subgroups (Figure 1C), suggesting that social rank is unlikely to affect contagion, although future experiments are needed to clarify this. Social hierarchy is not well expressed in the group behavior of adult rats with fetal valproate syndrome (Pelsűczi et al., 2020), so it is likely that even a high-ranked demonstrator rat would engage its cage-mates in collective actions. In an Alzheimer’s mouse model, mutant animals that were co-housed with wild-type cage-mates showed better copying and learning abilities compared to those housed separately (Kiryk et al., 2011). We did not observe such effects in the present experiment, although they may have been seen in studies with a larger number of healthy demonstrators. It is important to note that hypolocomotion is a behavioral trait of KM rats (Rebik et al., 2023; Rebik et al., 2022; Surina et al., 2011), which might contribute to their poor ability of rapid behavioral repetition (see red edges in Figures 1B, C). However, over the 30-min period analyzed, the group dynamics in KM observers differed from those in the control group (Figures 1B, C). Even the slowest animals had an opportunity to visit a water corner during this time. KM rats, overall, did not do this, resulting in a low number of activated observers (Figure 1C). Thus, hypolocomotion is unlikely to be the primary reason for the poor response seen in KM rats.

### 4.4 Lack of non-aversive tests for behavioral contagion

Most experiments studying behavioral contagion use emotionally negative procedures, such as painful stimuli or stressful environments, applied to demonstrators (Auer et al., 2024; Carnevali et al., 2020; Dimitroff et al., 2017; Qu et al., 2023). Stressful experiences are transmitted among conspecifics through ultrasonic vocalizations (Bruder et al., 2017; Brudzynski, 2013; Wöhr et al., 2015), olfactory stimuli (Kiyokawa et al., 2009; Raynaud et al., 2015; Takahashi et al., 2013), and other social interactions (Carnevali et al., 2020). Contagious yawning (Gallup, 2022; Massen and Gallup, 2017; Platek et al., 2005) is a rare example of spreading an emotionally neutral spontaneous behavior in animals. Thus, there is a lack of neutral experimental setups for studying social facilitation of learning and habituation.

A notable feature of our approach is its non-aversion. The present experimental paradigm could be applied to study, for example, social learning and intragroup hierarchy. Social learning and behavioral imitations are particularly deficient in patients with ASD. Negative emotional paradigms are often ethically unacceptable for research in human participants with ASD. Hence, emotional neutrality makes this paradigm particularly suitable for translation to human clinical tests.

### 4.5 Limitations and further directions

As a limitation of our study, one might point to a lack of any behavioral recording beyond the water corners. Unfortunately, the standard IntelliCage settings do not allow the recordings of additional behaviors, such as locomotion, vocalization, grooming, aggression, or play. However, in future specific add-on tools could be employed to assess these behavioral variables. Social interactions involve emotional exposure (Herrando and Constantinides, 2021). Emotional contagion is crucial in conspecific interaction, facilitating social learning, empathy, and group actions (Keysers et al., 2022; Pérez-Manrique and Gomila, 2022). Since deficient activation and behavioral copying were observed in the KM rat cohort, new experiments are needed to study the emotional states of participating animals. Neuropathological issues in KM rats, such as increased susceptibility to hemorrhagic stroke (Priezzhev et al., 2006) may contribute to their reduced social motivation. The impacts of genetic predisposition to convulsive epilepsies, of a history of seizure experiments, and of impaired interoception due to vascular accidents, on social contact deficits should be studied separately in future research. Additionally, caution is needed when extrapolating the pathological state of adult animals to model neurodevelopmental diseases of childhood. While adult animals offer more opportunities for basic and preclinical research and are widely used in practice, translating these results into developmental contexts requires additional steps.

Other psychosocial disorders, like self-harm or suicidal behavior, might have a contagious component (Seong et al., 2021). Therefore, a new quantitative approach to studying behavioral contagion in animals will be beneficial for preclinical and basic research beyond ASD neurobiology. Circadian fluctuations can have a significant impact on both locomotor and cognitive abilities (Kiryk et al., 2011), potentially introducing variability in experimental outcomes. Therefore, analyzing longer recording periods may offer a more comprehensive understanding of the underlying normal and deficient social processes.

The usage of only male rats, and the employment of only neurotypical demonstrator rats are the other limitations of the study. The female KM rats are not available from the breeder (Biological Faculty of Moscow State University), so the comparison of female individuals of Wistar and KM strains was not planned.

We used only Wistar demonstrator rats, to have the same conditions between the healthy and the KM groups of observers. It is not clear what would be the impact of “autistic” demonstrators on behavior of their group mates. It is quite possible that the individuals lacking social motivation would be outcasted by other group members. The group difference in contagion-induced water consumption warrants further study to distinguish between random factors and the formation of collective traits within a rat group. We hope that future experiments will clarify the points raised.

## 5 Conclusion

This study introduces an emotionally neutral paradigm for investigating behavioral contagion. Using the IntelliCage setup, we observed that healthy, non-water-deprived Wistar rats mimicked the behaviors of water-deprived conspecifics by visiting the drinking corners. Furthermore, they began to follow each other’s unmotivated visits, with peak behavior occurring in the first 30 min from the demonstrator’s reintroduction. In contrast, KM rats exhibited significantly reduced behavioral contagion, confirming their impaired social motivation and behavioral imitation.

Our novel approach, which employs a non-aversive experimental paradigm for studying behavioral contagion, provides a robust framework for exploring social behaviors in rodent models. This approach can allow a better understanding of social dysfunction and enables the development of therapeutic strategies for ASD and other psychosocial disorders.

## Data Availability

The original contributions presented in this study are included in this article/Supplementary material, further inquiries can be directed to the corresponding author.

## Footnotes

1
www.newbehavior.com

2
https://github.com/il0106/institute-of-higher-nervous-activity-and-neurophysiology/tree/main
