# Supplementary material for: Novel automated method to assess group dynamics reveals deficits in behavioral contagion in rats with social deficits
Source: Front Behav Neurosci. 2024 Dec 18;18:1519486. doi: 10.3389/fnbeh.2024.1519486 (PMC11688315; doi:10.3389/fnbeh.2024.1519486)
Supplement: Supplementary file 1 [file Table_1.docx]

**Supplementary Table 1**. The individual data for baseline and contagion-induced activity in rats and Wistar rats. For each individual rats the strain and IDs are given on the left columns. On the day of the behavioral contagion test, the time period from 8:50 to11:50 a.m. was used to establish a baseline of activity immediately prior to the presentation of the social stimulus (**BASELINE: Total Visits Number, Drinking Visit Number, Inspection Visit Number, Lick Contact Time)**. The contagion effects were assessed during the 30min following the re-introduction of the demonstrator rats **(CONTAGION: Total Visits Number, Drinking Visit Number, Inspection Visit Number, Lick Contact Time)**. All data were time-normalized: the three-hour baseline data sets were divided by 6 to match the duration of the 30-min contagion period.
