# Supplementary material for: Novel automated method to assess group dynamics reveals deficits in behavioral contagion in rats with social deficits
Source: Front Behav Neurosci. 2024 Dec 18;18:1519486. doi: 10.3389/fnbeh.2024.1519486 (PMC11688315; doi:10.3389/fnbeh.2024.1519486)
Supplement: Supplementary file 2 [file Table_2.docx]

**Supplementary Table 2.** A free software library, developed to visualize the activity of individual animals, and construct the dynamical graphs for groups

<https://github.com/il0106/Institute-of-Higher-Nervous-Activity-and-Neurophysiology/tree/main/IHNA_IntelliCage%20-%20Behavioral%20Contagion>
